# Reactivity and mechanism in chemical and synthetic biology

**DOI:** 10.1098/rstb.2022.0023

**Published:** 2023-02-27

**Authors:** Nigel G. J. Richards, Stephen L. Bearne, Yuki Goto, Emily J. Parker

**Affiliations:** ^1^ School of Chemistry, Cardiff University, Park Place, Cardiff CF10 3AT, UK; ^2^ Foundation for Advanced Molecular Evolution, 13709 Progress Boulevard, Alachua, FL 32615, USA; ^3^ Department of Biochemistry and Molecular Biology, Dalhousie University, 5850 College Street, Halifax, Nova Scotia, Canada B3H 4R2; ^4^ Department of Chemistry, Dalhousie University, 6274 Coburg Road, Halifax, Nova Scotia, Canada B3H 4R2; ^5^ Department of Chemistry, The University of Tokyo, 7-3-1 Hongo, Bunkyo-ku, Tokyo 113-0033, Japan; ^6^ Department of Chemistry, Victoria University of Wellington, Kelburn Parade, Wellington 6012, New Zealand

**Keywords:** aptamers, astrobiology, biosynthesis, enzymology, evolution, expanded genetic alphabets

## Abstract

Physical organic chemistry and mechanistic thinking provide a strong intellectual framework for understanding the chemical logic of evolvable informational macromolecules and metabolic transformations in living organisms. These concepts have also led to numerous successes in designing and applying tools to delineate biological function in health and disease, chemical ecology and possible alternative chemistries employed by extraterrestrial life. A symposium at the 2020 Pacifichem meeting was scheduled in December 2020 to discuss designing and exploiting expanded genetic alphabets, methods to understand the biosynthesis of natural products and re-engineering primary metabolism in bacteria. The COVID-19 pandemic led to postponement of in-person discussions, with the symposium eventually being held on 20–21 December 2021 as an online event. This issue is a written record of work presented on biosynthetic pathways and enzyme catalysis, engineering microorganisms with new metabolic capabilities, and the synthesis of non-canonical, nucleobases for medical applications and for studies of alternate chemistries for living organisms. The variety of opinion pieces, reviews and original research articles provide a starting point for innovations that clarify how complex biological systems emerge from the rules of chemical reactivity and mechanism.

This article is part of the themed issue ‘Reactivity and mechanism in chemical and synthetic biology’.

## 1. Introduction

Substantial advances in our understanding of how biomolecular structure underpins biological function have been made over the last half-century. Some of this progress has been driven by technological revolutions in computing [1], whole genome sequencing [2], mass spectrometry [3], analytical techniques for separating compounds from complex mixtures, microscopy [4] and molecular biology. It is also clear that synthetic chemistry has played a major role by providing small molecules capable of modifying or probing cellular metabolism, by confirming the structural assignments of secondary metabolites that mediate communication between plants, animals and microorganisms in ecological systems [5], and by discovering chemical reactions that are ‘orthogonal’ to those exploited by cells [6]. Indeed, the power of using such chemistries to interrogate biomolecular function within cells or to tag cellular components for purification and characterization was recognized by the award of the 2022 Nobel Prize in Chemistry to Bertozzi, Sharpless and Meldal. This fruitful intersection of Chemistry and Biology has been described by a bewildering variety of terms, of which Chemical Biology is now widely accepted within the scientific community. In this, and subsequent contributions, however, we limit the use of this term to the creation and application of chemical tools to solve specific biological problems. In a similar fashion, several definitions of what constitutes Synthetic Biology have appeared in the literature, which describe efforts to modify cellular metabolism to allow organisms to perform roles in sensing and the ‘green’ production of high-value compounds on a large scale. Without wishing to overlook a large amount of important work, here we limit our perspective of this field to the creation of artificial chemical systems that support (at least in principle) Darwinian evolution, which is the mechanism for creating life from non-living, organic and inorganic molecules [7]. Thus, non-natural components (i.e. compounds that are not present in living organisms on Earth) are created by chemical synthesis and then used to build systems that can evolve following the principles observed in living organisms. Such studies allow us to understand how complex biological entities and ecosystems can emerge from reactions that occur because of the electronic structure of molecules and the rules of chemical bonding. Perhaps as importantly, these studies provide insight into why naturally occurring organisms are constructed from biomolecular structures and chemistries in the ways that have survived on Earth as a result of Darwinian evolution.

## 2. Reactivity and mechanism in cellular metabolism and biosynthesis

The ability of living organisms to create diverse, structurally and stereochemically complex molecules from simple precursors has fascinated chemists for centuries. This synthetic versatility, which initially arose in primordial reducing conditions, must now be compatible with the presence of molecular oxygen and solvent water, both of which are reactive entities capable of interfering with chemical reactions. Indeed, an overwhelming number of organic chemical reactions require the exclusion of both oxygen and water to ensure high yields of product, although this situation is changing with our increasing knowledge of organocatalysis (recognized by the award of the 2021 Nobel Prize in Chemistry to List and MacMillan) [8]. Our ability to understand the chemical origins of cellular molecules and to delineate the chemistries used in their construction and transformation has been greatly facilitated by access to genome sequencing technology. Thus, bioinformatic analysis allows us to identify genes encoding the enzymes that mediate steps in the pathway leading to the molecule of interest, and to annotate their function on the basis of sequence homology and genome context [9]. Structural and mechanistic studies then allow us to delineate how each enzyme exerts regio- and stereochemical control, and substrate recognition, which subsequently informs inhibitor and drug development. The complexity of secondary metabolites often permits access to enzymes that catalyse transformations for which no efficient reagent, or sequence of steps, exists in organic chemistry. In these instances, protein engineering strategies (recognized by the award of the 2018 Nobel Prize in Chemistry to Arnold, Smith and Winter) can be exploited to obtain novel reagents capable of yielding high-value compounds using sustainable chemical processes [10].

**Figure 1.**
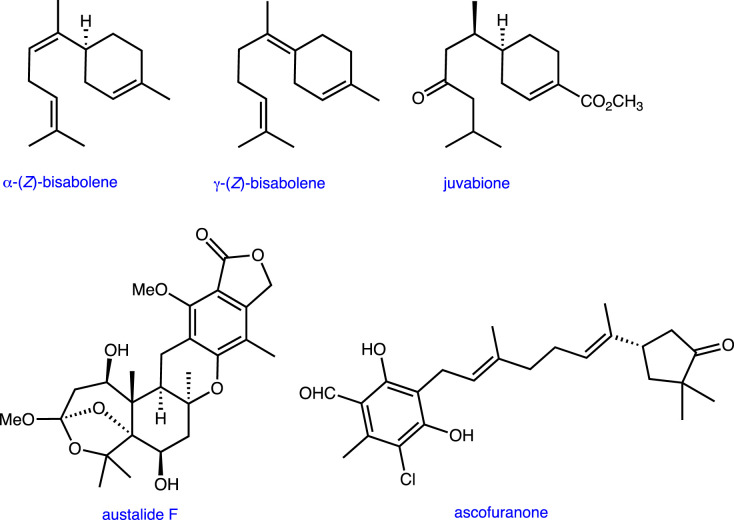
Selected terpenoid and meroterpenoid structures. (Online version in colour.)

For example, terpenoids represent the largest family of natural products and exhibit a breathtaking structural diversity of structures and biological activities [11]. A subset of these molecules, the sesquiterpenes, are constructed by cyclization and subsequent oxidative modifications from precursors that contain 15 carbon atoms. Many sesquiterpenes mediate inter-species interactions and their properties have driven the discovery of drugs, particularly those targeted against various types of cancer. In an original research paper, Hewage *et al*. [12] illustrate the power of genome-mining to identify genes encoding non-canonical terpene synthases and tailoring enzymes for the production of bisabolenes and their derivatives in fungi (figure 1). Importantly, the heterologous expression of enzymes encoded by these genes has provided tools for the combinatorial production of unnatural bisabolene analogues, which may exhibit novel bioactivities. Awakawa *et al*. [13] apply a similar approach to identify the gene cluster encoding the biosynthesis of austalide F. This compound is a meroterpenoid in which a polyketide synthase constructs an aromatic ring that can be alkylated with farnesyl pyrophosphate. Subsequent cyclization followed by oxidative ‘tailoring’ of the resulting polycyclic compound leads to austalide F. This study is important because other members of the meroterpenoid family, such as ascofuranone (anti-trypanosomiasis), are of significant interest to medicinal chemists (figure 1). Given that the chemical synthesis of meroterpenoids is not straightforward due to their stereochemical complexity, re-engineering the biosynthetic gene clusters encoding these natural products would expand access to novel analogues for subsequent screening.

Unfortunately, obtaining metabolic enzymes using heterologous expression systems provides only limited insights into their behaviour and kinetic properties in the host organism. This is a particular problem in studying non-ribosomal peptide synthetases (NRPSs), enzymes that mediate the ordered connection of amino acids (many of which are not the canonical amino acids present in proteins) into peptides with numerous biological activities [14]. Ishikawa *et al*. [15] address this important problem by conjugating photoactivatable groups onto an inhibitor of NRPS domains that activate phenylalanine for peptide incorporation. The resulting probes can then be used in photoaffinity labelling of NRPSs in lysates or bacterial cells. Importantly, the specificity conferred by the modified inhibitor prevents cross-linking to aminoacyl tRNA synthetases, and the speed of the NRPS domain labelling is fast enough to permit the use of short UV irradiation times. The study is, therefore, important because this new protocol obviates the need for long irradiation times that can cause damage, particularly to nucleobases, and therefore changes in cell metabolism.

**Figure 2.**
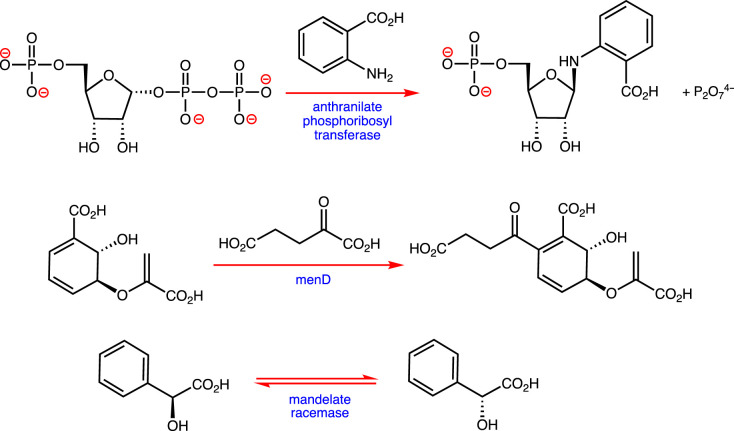
Reactions catalysed by anthranilate phosphoribosyl transferase, *Staphylococcus aureus* menD, and mandelate racemase. (Online version in colour.)

In addition to being of academic interest, many enzymes that mediate cellular metabolism represent drug targets [16,17]. The growing level of antibiotic resistance is, therefore, driving efforts to understand enzymes that catalyse reactions that are essential for bacterial survival but are absent in mammalian metabolism. The paper by Scully *et al*. [18] provides an introduction to such studies by reviewing the structural biology of anthranilate phosphoribosyl transferase. This enzyme catalyses a key step in the pathway leading from chorismate to tryptophan in microorganisms, fungi and plants (figure 2), and is considered to be a drug target because this reaction is essential for the growth of *Mycobacterium tuberculosis*. Inhibitors are likely to be useful in treating mycobacterial infections, and will address the growing problem of multi-drug resistance in *M. tuberculosis*. Building on this theme, Stanborough *et al*. [19] report new insights into the allosteric regulation of *Staphylococcus aureus* menD, the enzyme that catalyses the first committed step of the pathway leading to menaquinone (figure 2). As is widely known, this bacterium exhibits substantial levels of antibiotic resistance, and the discovery of drugs capable of targeting the allosteric sites of key metabolic enzymes represents a productive strategy for the development of new treatments for *S. aureus* and other ESKAPE microorganisms. Targeting menD, and perhaps other enzymes involved in menaquinone biosynthesis, is an especially promising approach given that humans do not employ menaquinones as electron carriers in respiratory chains.

Even when high-resolution structural information is available, obtaining inhibitors with high affinity and specificity for their enzyme target remains challenging. As pointed out many years ago by Wolfenden [20], stable molecules that resemble the transition state should exhibit sub-femtomolar dissociation constants. To date, however, few inhibitors with even picomolar affinities have been reported. Bearne [21] reviews the problems associated with designing these so-called transition state analogues, using mandelate racemase as a model system (figure 2). His analysis demonstrates the importance of maximizing the number of interactions within the active site in addition to ensuring that the electrostatic and steric properties of the molecule are similar to those calculated for the transition state.

Of course, even if new inhibitors are developed that are potent antibiotics, it is only a matter of time before bacterial resistance to these compounds will be seen [22]. Delineating the molecular mechanisms that give rise to resistance is, therefore, of the utmost importance when considering how to overcome this problem. Here, Lemay-St-Denis *et al*. [22] report a mechanism for the remarkable evolution of catalytic activity in a protein domain that is generally non-catalytic. Thus, drugs such as trimethoprim exert their effects by inhibiting dihydrofolate reductase (DHFR), an enzyme that is required in the metabolic cycle that converts UMP into TMP for subsequent DNA synthesis. This original research paper discusses how the family of type B DHFRs may have arisen from SH3 domains, which are employed in recognition and binding. Even though these enzymes have sub-optimal catalytic activity, their ability to reduce dihydrofolate is sufficient for bacteria to overcome the effects of trimethoprim, which only inhibits the type A DHFRs that are generally used. In addition to its importance for understanding resistance mechanisms, this study provides interesting insights into how enzymes might have been formed from non-catalytic precursors in early organisms.

## 3. Expanding the genetic alphabet

Using DNA and RNA to store and exploit information, respectively, is an essential feature of living organisms on Earth. In addition to showing the power of combining a small number of components to yield an essentially infinite pool of molecules, the ability of Watson–Crick nucleobases to undergo mutation underpins Darwinian evolution. Organisms, therefore, have the opportunity to develop novel chemistries, and molecular mechanisms that regulate cellular metabolism and communication thereby enhancing their biological fitness. The ubiquity of Watson–Crick nucleobases through biology, however, raises the question of whether such structures represent the only molecular solution to the problem of storing and using information in living organisms [23,24]. Moreover, if DNA and RNA are uniquely suited for the appearance of life, what are the chemical properties of these macromolecules that permit them to adopt structures through which they can exert their biological functions? If alternate genetic systems exist, however, can they be constructed by chemical synthesis and made to function in organisms that have evolved under the constraints imposed by the environment of our planet? Indeed, can we use our understanding of physical organic chemistry to build novel nucleobases that can increase the information density of these informational macromolecules?

**Figure 3.**
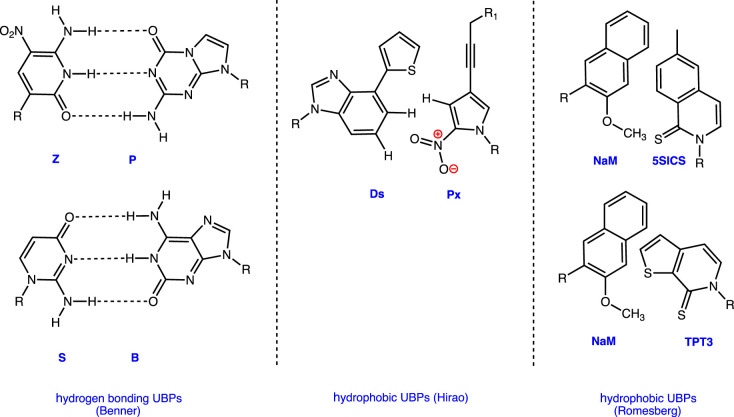
Structures of non-canonical nucleobases used in present-day expanded genetic alphabets. (Online version in colour.)

**Figure 4.**
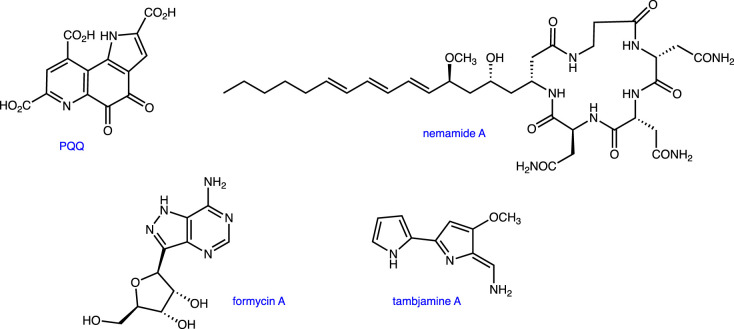
Structures of other natural products discussed at the virtual symposium. (Online version in colour.)

Answers to these questions are provided by a series of papers in this issue. In the first of these, Benner [25] provides a comprehensive review of this field, particularly his own efforts over the past three decades to develop non-canonical nucleobase pairs that can augment those present in DNA and RNA. Not only has this work clarified the molecular features needed for informational molecules in biology but it has also provided an intellectual framework for technologies to identify life elsewhere in the Universe, which may be based on alternate genetic systems (figure 3). In an accompanying original research paper, Shukla *et al*. [26] describe the structural characterization of ‘ALIEN’ DNA containing the nucleobases developed by Benner [25]. These studies show that DNA can be built using only these non-canonical nucleobases and reveal how the DNA helix exhibits sequence-specific structural properties, which could be exploited by DNA-binding proteins to control transcription. In addition, it appears that ALIEN DNA can adopt a wider range of B-form structures than DNA constructed with only Watson–Crick nucleobases. Numerous studies have shown that the presence of non-canonical nucleobases can improve the ability of aptamers to bind to their cellular targets and the implications of these observations for nanotechnology and the development of nucleic acid therapeutics are summarized in an opinion piece by Wang *et al*. [27] These authors point out the large chemical diversity of aptamers and the fact that base-pairing can be exploited to control their three-dimensional structure in a precise manner. These ideas are well illustrated in the subsequent research article by Kimoto *et al*. [28] who exploit a different type of non-canonical nucleobase, which is hydrophobic and lacks hydrogen bonding groups (figure 4), to obtain aptamers with improved affinities and selectivities. Remarkably, the introduction of only one non-canonical nucleobase allows the aptamers to adopt well-defined tertiary structures.

Expanded genetic alphabets are also impacting synthetic biology, as summarized in the review by Romesberg [29] . He and his co-workers summarize how a shape complementary nucleobase pair (figure 3) is being exploited in the creation of new codon/anti-codon pairs, which can be used to express proteins in *Escherichia coli* containing amino acids that are not found in Nature. Once again, Romesberg's successful production of ‘semi-synthetic organisms’ has profound implications for alternative metabolic pathways that might be used by non-terrestrial life, should it exist. In addition, access to these technologies opens the door to obtaining new types of protein-based therapeutics and enzymes for use in the sustainable industrial synthesis of bulk chemicals (‘green’ chemistry). Of course, routine access to proteins containing non-proteinaceous amino acids depends on a detailed understanding of how aminoacylated tRNAs interact with EF-Tu, the protein that positions these molecules on the ribosome. Original research by Katoh & Suga [30] shows the importance of this protein/nucleic acid interaction for the incorporation efficiency of non-proteinaceous amino acids, and shows that the key to producing proteins containing d-α-amino acids is to create EF-Tu variants and modified tRNAs for which they exhibit enhanced binding affinity. The work of Katoh and co-workers thereby provides important new insights into how protein expression can be manipulated, with its implications for protein production in semi-synthetic organisms possessing expanded genetic alphabets. The final paper, by Hasan *et al*. [31], in this issue also discusses original research concerning the molecular processes that take place during ribosomal protein synthesis. Thus, some tRNA variants appear to increase the mis-incorporation of amino acids and to inhibit protein synthesis, which affects protein homeostasis in mammalian cells. This observation also has significant implications for obtaining novel proteins through the use of tRNAs engineered to possess anti-codons built from non-canonical nucleobases, which can carry non-canonical amino acids to the ribosome.

## 4. Other topics

The virtual Symposium on Reactivity and Mechanism in Chemical and Synthetic Biology included a number of interesting presentations that are not represented by papers in this collection. We especially want to note the talks given by three early career researchers making important contributions to this field as independent investigators. Thus, Graeme Howe discussed computational studies of how enzymes can manipulate P–H bonds, Wen Zhu highlighted the importance and role of auxiliary FeS clusters in PqqE (a radical SAM enzyme that catalyses an unusual cross-linking reaction in PQQ biosynthesis) [32] and Yutaro Saito presented his work on novel probes for the *in vivo* detection of peptidase activity [33]. Other talks described the elucidation and chemical logic of biosynthetic pathways leading to the C-nucleoside formycin A (Nigel Richards) [34], and nemamides, which are novel hybrid polykeptide/non-ribosomal peptide natural products (Rebecca Butcher) [35]. Continuing this theme, Avena Ross outlined how she is employing genome analysis to identify and understand the evolutionary history of pathways leading to naturally occurring poly-pyrroles such as the tambjamines (figure 3) [36].

Several other speakers highlighted new work in mechanistic enzymology and inhibitor design, including how conformational dynamics modulate enzyme activity (Colin Jackson) [37], and the importance of Fe-dependent chemistry in the metabolism of phosphorus–carbon covalent bonds (David Zechel) [38]. Recent work on the application of terpene synthases in the biocatalysis of modified terpenes was also discussed by Luke Johnson [39], Andrew Murkin described reactivity-based strategies to identify potent, specific enzyme inhibitors [40], and John Pezacki presented an activity-based method for the functional profiling of microRNA targets. Finally, Piet Herdewijn gave a fascinating overview of his wide-ranging efforts to create highly modified variants of DNA and RNA [41], with the goal of developing new information-rich macromolecules.

## 5. Some final thoughts

The COVID-19 pandemic and its aftermath have delayed this special issue, and we thank our contributors for their forebearance. Even so, the insights contained in these papers are likely to drive the development of this field in the coming years, especially given the comprehensive surveys of work with novel nucleobases and their applications in medicine and synthetic biology. We are proud to have produced an issue that reflects the excitement of our virtual symposium at Pacifichem 2021, and we very much appreciate the efforts of the editorial staff at the Royal Society. In particular, Helen Eaton has shown unflinching patience with us as we assembled the manuscripts included in this issue. Finally, we must thank our international groups of authors for their generosity in providing their insights into key problems associated with chemical and synthetic biology.

## Data Availability

This article has no additional data.
